# Medical Reversals, Spins e Resultados Divergentes em Ensaios na Cardiologia

**DOI:** 10.36660/abc.20240884

**Published:** 2025-07-29

**Authors:** José Nunes de Alencar, Bruno Robalinho Cavalcanti, Guilherme Augusto Teodoro Athayde

**Affiliations:** 1 Instituto Dante Pazzanese de Cardiologia São Paulo SP Brasil Instituto Dante Pazzanese de Cardiologia, São Paulo, SP – Brasil; 2 Universidade Federal de Campina Grande Campina Grande PB Brasil Universidade Federal de Campina Grande, Campina Grande, PB – Brasil; 3 Dom José Maria Pires Metropolitan Hospital João Pessoa PB Brasil Dom José Maria Pires Metropolitan Hospital, João Pessoa, PB – Brasil

**Keywords:** Cardiologia Baseada em Evidências, Reversões Médicas, Viés de *Spin*

## Abstract

A medicina cardiovascular tem testemunhado avanços notáveis, mas mesmo intervenções altamente conceituadas podem ser prejudicadas por raciocínios falhos, suposições mecanicistas excessivas e a apresentação dos dados de maneira seletiva. Este artigo examina armadilhas cruciais na cardiologia contemporânea, como *medical reversals*, o impacto do *spin* e como os métodos bayesianos podem oferecer maior clareza na avaliação de evidências, ao integrar conhecimento prévio com novos dados para gerar conclusões mais probabilísticas e baseadas no contexto. Esta revisão defende uma abordagem crítica e ponderada para avaliação de pesquisas, alertando os cardiologistas sobre os perigos de aceitar conclusões de estudos sem questionar. A adoção dessa postura vigilante ajudará a garantir que as terapias e intervenções emergentes realmente melhorem os resultados dos pacientes, orientando os médicos em direção a estratégias mais confiáveis, transparentes e benéficas no campo da medicina cardiovascular, em constante evolução. Essa vigilância é importante para preservar a integridade da investigação científica e o progresso significativo no cuidado ao paciente. Essa abordagem promove a confiabilidade dos dados publicados.

## Graphical abstract

**Figura Central:**
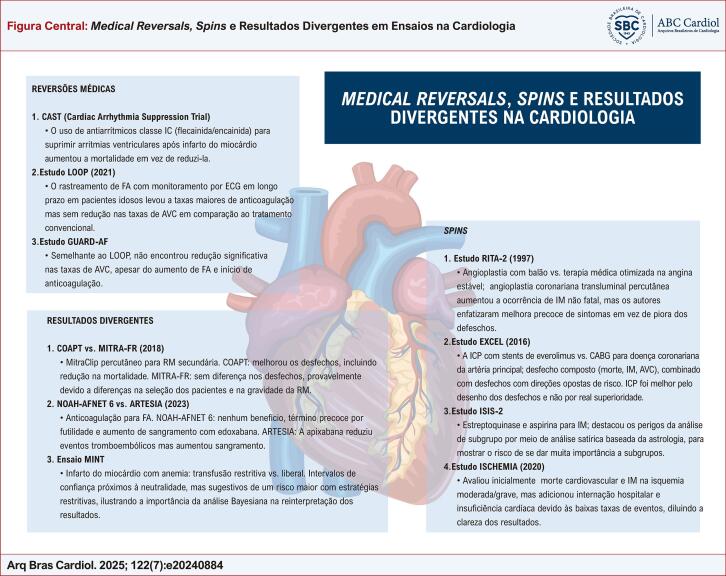
*Medical Reversals, Spins* e Resultados Divergentes em Ensaios na Cardiologia

## Introdução

Nas últimas décadas, a medicina cardiovascular testemunhou progressos notáveis, apoiados por rigorosos ensaios clínicos e diretrizes abrangentes. Esses avanços melhoraram, de forma inequívoca, os resultados dos pacientes, reduzindo a mortalidade e aumentando sua qualidade de vida. No entanto, essa evolução científica não esteve livre de erros, controvérsias e reviravoltas surpreendentes que desafiam nossas noções preconcebidas de fisiopatologia e a eficácia de certas intervenções. Suposições mecânicas que antes pareciam evidentes cederam sob o escrutínio de evidências robustas, ilustrando os perigos de confiar apenas na lógica sem testar hipóteses por meio de desenhos de estudo rigorosos. Intervenções iniciais que prometiam melhorar a sobrevivência com base apenas na plausibilidade mecanicista, por vezes, levaram a reviravoltas clínicas inesperadas quando submetidas a investigações rigorosas.^1,2^

A importância da avaliação crítica e da interpretação dos dados clínicos na cardiologia não pode ser subestimada. Os médicos precisam navegar em um panorama cada vez mais complexo de evidências médicas, que inclui estudos com resultados conflitantes, possíveis distorções nos resultados relatados, e padrões de rigor metodológico em evolução. Avaliar a validade, aplicabilidade e relevância clínica de novos dados tornou-se uma habilidade essencial para o cardiologista moderno.^3^ Tal avaliação crítica promove decisões individualizadas e adaptadas, seja considerando os riscos e benefícios de intervenções coronarianas, selecionando candidatos para ablação por cateter de fibrilação atrial (FA) ou determinando quando iniciar terapias avançadas para insuficiência cardíaca (IC).^4^

Este artigo examina como a dependência em suposições mecanicistas não testadas, a suscetibilidade à distorção e os desafios na interpretação de resultados complexos ou conflitantes podem comprometer a integridade da cardiologia baseada em evidências. Com base em casos emblemáticos, destaca como reviravoltas médicas, manipulação de desfechos, e a apresentação seletiva de resultados podem distorcer a tomada de decisão clínica (Figura Central e Tabela 1).

**Tabela 1:** – Definições chave: reversões médicas, *spin*, e resultados divergentes

Termo	Definição	Considerações chave /exemplos
*Medical reversals* ou reversões médicas	Ocorrem quando uma intervenção clínica amplamente aceita – geralmente baseada incialmente em estudos observacionais ou racional mecanicista – é posteriormente refutada com base em ensaios clínicos randomizados	Por exemplo, o uso inicial de antiarrítmicos da classe IC para suprimir arritmias ventriculares foi refutado pelo ensaio CAST, que demonstrou aumento na mortalidade.
*Spin*	Refere-se à apresentação seletiva ou ao enquadramento dos resultados de estudos para acentuar desfechos favoráveis, enquanto minimiza ou obscurece achados adversos ou neutros.	Um exemplo é o estudo RITA-2, onde a melhora sintomática precoce foi destacada para a angioplastia coronariana transluminal percutânea, apesar do desfecho composto geral favorecer a terapia médica otimizada.
Resultados divergentes	Descreve situações em que estudos de alta qualidade que investigam a mesma questão clínica geram desfechos conflitantes, em geral por diferenças no delineamento, populações ou definições de desfechos.	Um claro exemplo inclui os resultados contrastantes do COAPT e do MITRA-FR avaliando o uso do MitraClip para regurgitação mitral funcional.

### Nem tudo é como parece: reversões médicas na cardiologia

A cardiologia está repleta de estudos que desafiam expectativas lógicas e destacam como a medicina baseada em evidências pode reverter o que antes parecia ser verdades indiscutíveis.^5,6^ Um exemplo marcante está no tratamento de arritmias ventriculares após infarto agudo do miocárdio (IAM). Nas décadas de 1970 e 1980, a criação de unidades de cuidados coronarianos reduziu drasticamente a mortalidade hospitalar de pacientes com IAM.^7^ Posteriormente, tornou-se evidente que muitos sobreviventes passaram a apresentar arritmias ventriculares, que foram identificadas em várias investigações como preditores de mortalidade.^8^ Com base nesse entendimento teórico, uma equipe de pesquisadores nos Estados Unidos propôs que as taxas de sobrevivência poderiam ser melhoradas para pacientes com miocardiopatia isquêmica que apresentavam alto risco de morte por arritmias ventriculares, caso esses batimentos cardíacos irregulares fossem suprimidos. O primeiro passo na avaliação dessa hipótese foi o Estudo Piloto de Arritmia Cardíaca (CAPS, *Cardiac Arrhythmia Pilot Study*), conduzido em 1986. Este estudo demonstrou que os medicamentos antiarrítmicos da classe IC, que funcionam como bloqueadores de canais de sódio, eram capazes de praticamente eliminar as arritmias ventriculares nesses indivíduos.^9^ Com isso, parecia lógico adicionar esses agentes ao arsenal terapêutico; se as arritmias pudessem ser suprimidas, a mortalidade deveria ser reduzida.

No entanto, o Estudo de Supressão de Arritmia Cardíaca (CAST, *Cardiac Arrhythmia Suppression Trial*) contrariou essas suposições. O estudo incluiu 1498 pacientes randomizados para receber flecainida/encainida ou placebo. Após 10 meses, o ensaio foi interrompido devido ao excesso de mortes — especialmente mortes arrítmicas — no grupo que recebeu os medicamentos antiarrítmicos. Essa reversão tornou-se uma das mudanças científicas mais marcantes da época e limitou drasticamente o uso de antiarrítmicos da classe IC em pacientes com doença cardíaca estrutural, devido ao seu potencial pró-arrítmico.^10^

Outro achado inesperado foi observado no contexto da triagem e tratamento da FA. A FA é a arritmia sustentada mais comum na prática clínica e está associada à mortalidade e eventos tromboembólicos (ETs), particularmente acidente vascular cerebral (AVC) e IC. Além disso, a anticoagulação pode reduzir significativamente e com segurança o risco de ETs.^11^ Parecia evidente que a identificação e a anticoagulação precoces em pacientes com FA resultariam em benefícios inequívocos. No entanto, o estudo LOOP, publicado em 2021, desafiou essa ideia.^12^ Nesse ensaio, 6205 pacientes com idades entre 70 e 90 anos, com pelo menos um fator de risco adicional para AVC, foram randomizados para o manejo convencional ou monitoramento eletrocardiográfico subcutâneo contínuo e de longo prazo para a detecção de FA. Pacientes com episódios de FA com duração igual ou maior que seis minutos iniciaram anticoagulação. Após mais de cinco anos, o diagnóstico de FA foi três vezes mais comum no grupo monitorado, e a anticoagulação foi iniciada com maior frequência. No entanto, não houve diferenças nas taxas de AVC ou sangramento entre os grupos. Os resultados do estudo estão alinhados com os de outros ensaios, como o GUARD-AF, que também não encontraram redução significativa nas taxas de AVC com a triagem de FA, sugerindo que nem toda FA detectada pode ser clinicamente significativa o suficiente para justificar a anticoagulação.^13^

Uma crítica é que a população do estudo LOOP pode não ter sido selecionada de forma ideal para se beneficiar da triagem de FA. As análises de subgrupos sugeriram benefícios potenciais em populações específicas, como aquelas sem doença cardiovascular prévia, mas esses achados não foram definitivos e exigem investigação adicional.^14^ Além disso, preocupações foram levantadas sobre a metodologia do estudo e a decisão de iniciar anticoagulação para episódios de FA com duração de apenas seis minutos ou mais, já que permanece incerto se episódios tão breves requerem tratamento.

Em relação a outra reviravolta médica importante, estudos observacionais iniciais sugeriram que a terapia de reposição hormonal (TRH) para mulheres após a menopausa poderia reduzir o risco cardiovascular. Esses estudos indicaram potenciais vantagens, incluindo a diminuição da doença arterial coronariana (DAC) e das taxas de mortalidade.^15^ Ensaios clínicos randomizados, notadamente o WHI (*Women’s Health Initiative*),^16^ contradisseram esses achados ao mostrar um aumento no risco de eventos cardiovasculares, incluindo DAC e AVC, em mulheres que recebiam TRH.^16^ Os ensaios WHI foram interrompidos precocemente devido a esses riscos aumentados, levando a uma mudança significativa na prática clínica, e não se utilizando a TRH para proteção cardiovascular.^17^Investigações mais recentes revelaram que, para mulheres que entraram na menopausa nos últimos 10 anos, a TRH oferece vantagens terapêuticas sem aumentar os perigos cardiovasculares. Ensaios clínicos^18^ e meta-análises^19^ forneceram evidências que apoiam essas observações. Este grupo se enquadra na “janela de oportunidade” para a prescrição de TRH, que tem sido recomendada por diretrizes internacionais e nacionais, particularmente para mulheres sem alto risco ou eventos cardiovasculares prévios. Essas diretrizes recomendam que a TRH seja iniciada dentro de 10 anos após a menopausa e/ou antes dos 60 anos de idade; iniciar a terapia após os 60 anos ou mais de 10 anos após a menopausa pode elevar o risco absoluto de eventos adversos cardiovasculares.^20,21^

### Interpretações distorcidas: *spin* na pesquisa cardiovascular

Alguns estudos tiveram suas conclusões sutilmente distorcidas pelos próprios autores, lançando uma luz mais favorável sobre um determinado tratamento do que os dados justificam. Essa prática, conhecida como *spin*, pode enganar leitores e clínicos, influenciando potencialmente as decisões terapêuticas.^22^

Um exemplo notável de *spin* pode ser encontrado no contexto da DAC. O estudo RITA-2, publicado em 1997, randomizou 1018 pacientes com angina estável para angioplastia com balão – angioplastia transluminal percutânea (PTCA, *percutaneous transluminal coronary angioplasty*) ou terapia médica otimizada (TMO).^23^ O desfecho composto primário — morte por qualquer causa e infarto do miocárdio (IM) não fatal — foi avaliado ao longo de cinco anos (seguimento médio de 2,7 anos). Os resultados mostraram que a PTCA aumentou o risco do desfecho composto primário em comparação com a TMO, impulsionado principalmente por IM não fatal. Embora ambos os grupos tenham inicialmente experimentado melhora nos sintomas, a significância estatística a favor da PTCA sobre a TMO diminuiu após três anos. No entanto, os autores enfatizaram uma melhora precoce nos sintomas nos pacientes submetidos à PTCA e sugeriram que os clínicos ponderassem esse benefício contra um “pequeno” risco excessivo do procedimento. Essa abordagem não refletiu com precisão o desfecho primário, no qual a PTCA piorou os resultados. Com um número necessário para prejudicar de 33 em 2,7 anos, o *spin* do estudo desviou a atenção do resultado negativo do desfecho primário.

Um exemplo semelhante surgiu com o estudo EXCEL, publicado em 2016, que avaliou a intervenção coronariana percutânea (ICP) com *stents* de segunda geração liberadores de everolimus em comparação com a cirurgia de revascularização do miocárdio (CABG) para doença da artéria coronária principal esquerda de complexidade anatômica baixa a intermediária.^24^ O desfecho composto primário — morte, IM e AVC — foi metodologicamente questionável. Sabe-se que a CABG aumenta o risco de acidente vascular cerebral em comparação com a PCI, enquanto a PCI pode estar associada a taxas mais altas de IM espontâneo. Combinar esses eventos em um único desfecho composto significava que as duas intervenções puxavam os resultados em direções opostas. Também surgiram preocupações sobre o uso da análise por intenção de tratar em vez do protocolo, dado o cruzamento substancial, e as definições em evolução de IM durante o estudo. Tais mudanças podem ter favorecido a PCI. Como resultado, uma abordagem mais transparente separaria o IM relacionado ao procedimento e o AVC como desfechos de segurança e focaria exclusivamente no IM espontâneo como desfecho primário, como o estudo NOBLE eventualmente fez.^25^

Análises de subgrupos representam outra via comum para *spin*. O estudo ISIS-2, um ensaio clínico randomizado, controlado por placebo e com desenho fatorial 2x2, com mais de 17 000 pacientes, testou estreptoquinase e um mês de aspirina a 162,5 mg/dia no contexto de IAM.^26^ O estudo demonstrou uma redução de 20% na mortalidade vascular com aspirina, 23% com estreptoquinase e uma redução de 40% com a combinação de ambos ao longo de cinco semanas. A redução absoluta do risco com o uso de aspirina sozinha foi de 2,4 mortes por 100 pacientes tratados, correspondendo a um número necessário para tratar de 42. Apesar desses resultados impactantes, os editores de revistas na época exigiram análises de subgrupos. Em uma resposta deliberadamente irônica, os autores relataram resultados por signos astrológicos, descobrindo, por exemplo, que pacientes nascidos sob Gêmeos e Libra aparentemente não se beneficiaram da aspirina. Essa demonstração bem-humorada destacou a insensatez de confiar excessivamente em achados arbitrários de subgrupos e enfatizou a necessidade de interpretar essas análises com ceticismo.^27^

Outro meio sutil, mas impactante, de moldar a narrativa de um ensaio está na alteração dos desfechos predefinidos ou suas definições durante a investigação. O estudo ISCHEMIA é um exemplo emblemático. Este estudo de referência, com custo aproximado de 100 milhões de dólares, foi publicado em 2020 e incluiu 5179 pacientes com isquemia moderada a grave, randomizando-os para TMO sozinha ou TMO mais cateterismo cardíaco seguido de ICP, se indicado.^28^ Inicialmente, o desfecho composto primário era morte cardiovascular e IM. No entanto, devido à incidência menor que a esperada desses eventos, os investigadores adicionaram hospitalização e IC ao desfecho composto primário.

Embora os investigadores tenham definido essa modificação *a priori*, a decisão acabou enfraquecendo a clareza interpretativa do estudo. A incorporação de desfechos subjetivos, tais como angina instável ou hospitalizações por IC, juntamente com desfechos objetivos, como morte e IM, diluiu a clareza e a confiabilidade das conclusões. Os resultados do estudo ISCHEMIA demonstraram que não houve diferença significativa entre as estratégias invasivas e conservadoras, reforçando o entendimento de que, mesmo em pacientes com isquemia moderada ou grave, a DAC estável frequentemente apresenta um prognóstico favorável a longo prazo.^29^

A seleção de desfechos — e suas suposições subjacentes — pode criticamente moldar os resultados de um estudo. Considere o estudo OPTION, recentemente introduzido, e patrocinado pelo fabricante de um dispositivo usado para oclusão do Apêndice Atrial Esquerdo (AAE) juntamente com ablação de FA.^30^ O estudo OPTION compara ablação de FA mais AAE concomitante à ablação de FA mais anticoagulantes orais diretos contínuos. À primeira vista, o objetivo do estudo — potencialmente reduzir ou eliminar a necessidade de anticoagulação a longo prazo — pode atrair pacientes e clínicos. No entanto, uma análise mais detalhada do design do OPTION levanta várias preocupações. O estudo emprega um modelo de não inferioridade e inclui mortalidade por todas as causas, um desfecho conhecido por permanecer inalterado tanto pela ablação quanto pelo AAE, como parte de uma medida de eficácia composta. Essa abordagem, juntamente com um tamanho de amostra relativamente pequeno e uma margem de não inferioridade escolhida com base em suposições otimistas de taxa de eventos, facilita que o AAE alcance a não inferioridade sem demonstrar vantagens significativas na prevenção de AVC ou embolia sistêmica. Outras preocupações surgem da escolha de conduzir a análise primária na população por intenção de tratar — menos apropriada para estudos de não inferioridade — e excluir sangramentos procedimentais do desfecho primário de segurança, subestimando assim os verdadeiros riscos procedimentais.^31^

### Reconciliando resultados divergentes em ensaios clínicos

Às vezes, ensaios clínicos de alta qualidade produzem resultados conflitantes sobre a mesma questão médica, tornando difícil para os profissionais de saúde determinarem a melhor forma de aplicar esses achados na prática.

Um caso representativo envolve a reparação percutânea da regurgitação mitral secundária, usando o dispositivo MitraClip. Dois ensaios clínicos de referência, o COAPT^15^ e o MITRA-FR,^16^ ambos publicados em 2018, examinaram o impacto clínico de se adicionar o MitraClip à terapia médica orientada por diretrizes em pacientes com regurgitação mitral funcional. Enquanto o COAPT tenha focado na hospitalização por IC em um ano, o MITRA-FR avaliou o desfecho composto de morte e hospitalização por IC em um período de tempo semelhante. Apesar de metodologias similares, os resultados divergiram significativamente. No COAPT, o MitraClip reduziu significativamente as hospitalizações e a mortalidade geral, enquanto o MITRA-FR não mostrou melhorias. Quando ensaios clínicos produzem resultados divergentes, é essencial realizar uma análise aprofundada para entender as razões subjacentes a essas discrepâncias. Vários fatores-chave devem ser examinados:

1. **Delineamento e população do estudo**: Diferenças no desenho do estudo, tais como critérios de inclusão e exclusão, podem levar a variações nas populações de pacientes. Por exemplo, o COAPT e o MITRA-FR tiveram critérios diferentes para a gravidade da regurgitação mitral e função ventricular esquerda, o que influenciou significativamente seus resultados.^32^
2. **Definições de desfechos**: Variações na forma como os desfechos primários e secundários são definidos e medidos podem levar a diferentes interpretações de eficácia. O estudo COAPT teve critérios mais rigorosos para o sucesso do procedimento e durabilidade da redução da regurgitação mitral em comparação ao MITRA-FR.^32^
3. **Protocolos de tratamento**: Diferenças na implementação dos protocolos de tratamento, incluindo o uso da terapia médica orientada por diretrizes, podem afetar os resultados dos estudos. O COAPT garantiu que os pacientes estivessem recebendo a terapia médica máxima tolerada antes da inscrição, o que não foi rigorosamente aplicado no MITRA-FR.^32^
4. **Experiência do centro e dos operadores**: A experiência dos centros e dos operadores que realizam as intervenções pode impactar as taxas de sucesso do procedimento e os resultados. O COAPT foi conduzido em centros com significativa experiência na reparação percutânea da valva mitral, o que pode ter contribuído para seus resultados positivos.^32^
5. **Análise estatística e interpretação**: Os métodos estatísticos usados para analisar os dados, incluindo dados falante e ajustes quanto a comparações múltiplas, podem influenciar os resultados.^33^

Um desafio similar surgiu em 2023 com dois ensaios avaliando a anticoagulação para FA subclínica detectada por dispositivos cardíacos implantáveis. O NOAH-AFNET 6 incluiu 2356 pacientes com idade ≥65 anos com um ou mais episódios de FA subclínica com duração de seis minutos ou mais, além de um fator de risco tromboembólico adicional, randomizando-os para placebo ou edoxabana.^34^ O desfecho primário foi um composto de morte cardiovascular, AVC ou embolia sistêmica. No ARTESIA, 4012 pacientes com pelo menos um episódio de FA subclínica com duração de seis minutos ou mais (mas ≤24 horas), foram randomizados para apixabana ou aspirina (81 mg) e avaliados quanto à incidência de AVC ou embolia sistêmica.^35^ O NOAH-AFNET 6 foi interrompido precocemente por futilidade, sem diferença no desfecho primário e com aumento de sangramentos no grupo edoxabana. No ARTESIA, a apixabana reduziu ETs, mas com taxas mais altas de sangramento. Ao examinar as possíveis causas desse resultado divergente, podemos concluir que:

1. **Delineamento e população do estudo**: O NOAH-AFNET 6 focou em pacientes com episódios de alta frequência atrial, detectados por dispositivos eletrônicos cardíacos implantáveis, utilizando edoxabana como anticoagulante. O ARTESIA avaliou a apixabana em uma população semelhante, mas incluiu pacientes com fatores de risco adicionais para AVC, como uma maior prevalência de doença vascular. O estudo ARTESIA também teve um tamanho de amostra maior, incluindo 4012 pacientes em comparação com 2534 no NOAH-AFNET 6.^36^
2. **Definições de desfechos**: Ambos os estudos definiram seu desfecho primário de eficácia como um composto de AVC, embolia sistêmica, IM, embolia pulmonar ou morte cardiovascular. No entanto, o ARTESIA incluiu ataque isquêmico transitório com evidência de infarto cerebral em ressonância magnética ponderada por difusão como parte de seu desfecho primário, o que não foi explicitamente mencionado no NOAH-AFNET 6.^37^
3. **Protocolos de tratamento**: Os protocolos de tratamento foram diferentes quanto à escolha do anticoagulante – edoxabana no NOAH-AFNET 6 e apixabana no ARTESIA. No NOAH, edoxabana foi comparada a placebo, e no ARTESIA, à aspirina.

Quando os dados são divergentes, uma meta-análise pode ajudar. Uma meta-análise de dois ensaios demonstrou que a anticoagulação oral reduziu o risco de AVC isquêmico (RR 0,68, IC95% 0,50-0,92) e aumentou o risco de sangramentos maiores (RR 1,62; IC 95% 1,05-2,50). A meta-análise também destacou uma baixa heterogeneidade (I^2^ = 0%), indicando consistência nos achados entre os ensaios.^38^

Outra abordagem para reconciliar resultados divergentes e aprimorar a interpretação de ensaios clínicos reside no raciocínio Bayesiano. Métodos Bayesianos integram crenças prévias com novas evidências adquiridas para gerar probabilidades posteriores, proporcionando uma interpretação mais intuitiva e clinicamente relevante em comparação à estatística frequentista tradicional. Ao focar na probabilidade de que uma determinada intervenção seja realmente benéfica, em vez de confiar apenas em valores de p ou em limites binários de significância, o pensamento Bayesiano permite uma avaliação mais flexível e sensível ao contexto das evidências.^3^

A estatística Bayesiana começa com uma probabilidade prévia de que um dado tratamento é eficaz.^39^ No contexto de um ensaio clínico randomizado comparando uma nova Terapia (T) com um Controle (C), a suposição inicial pode ser de equilíbrio: P(T > C) = 0,5. Após coletar e analisar os dados do estudo, os resultados observados são combinados com essa probabilidade anterior para gerar uma probabilidade posterior. Se a probabilidade posterior de que T seja superior a C ultrapassar um determinado limite (por exemplo, 0,975), pode-se ter mais confiança de que a intervenção é genuinamente eficaz.^40^No entanto, se os dados sugerirem nenhuma vantagem significativa, o framework Bayesiano expressa prontamente como esses resultados modificam a crença na eficácia do tratamento. Diferentemente dos métodos frequentistas, em que um intervalo de confiança de 95% não atribui uma probabilidade ao parâmetro de interesse, o intervalo de credibilidade Bayesiano de 95% fornece uma declaração direta de probabilidade sobre o parâmetro.^41^ Em vez de afirmar que um resultado é estatisticamente significativo, os métodos Bayesianos permitem que os médicos discutam a probabilidade de que uma diferença nos desfechos (por exemplo, uma redução de IM ou AVC) seja real e clinicamente relevante. Dessa forma, o raciocínio Bayesiano vai além dos valores de p, direcionando interpretações mais aplicáveis que podem orientar a tomada de decisões centrada no paciente.

Um bom exemplo de como a análise Bayesiana pode oferecer uma interpretação distinta dos resultados é sua aplicação no estudo MINT. Este estudo incluiu 3504 pacientes com IM e anemia (hemoglobina <10 mg/dL), que foram randomizados para estratégias restritivas de transfusão – transfundindo apenas quando os níveis de hemoglobina estavam abaixo de 7-8 mg/dL – ou estratégias liberais (transfundindo quando a hemoglobina <10 mg/dL). O desfecho primário, um composto de IM e morte em 30 dias, foi semelhante entre os grupos, com um intervalo de confiança de 0,99 a 1,34; p=0,07. Apesar disso, houve uma forte tendência para um maior número de eventos no grupo restritivo (o que poderia ter sido confirmado com um tamanho de amostra maior), incluindo tanto mortes quanto IM, juntamente com uma taxa muito baixa de eventos adversos associados à transfusão. Diante desses achados, analisar os resultados sob a perspectiva do equilíbrio entre benefício e risco da estratégia nesta população — com base no conhecimento existente — é crucial para identificar subgrupos de pacientes que poderiam se beneficiar da transfusão, mesmo no contexto de um estudo “negativo”.^42^

Nas análises frequentistas tradicionais, a significância estatística é geralmente determinada por um valor de p inferior a 0,05. Essa abordagem pode levar a interpretações binárias, “positivo vs. negativo”, negligenciando toda a distribuição de probabilidade do efeito verdadeiro. Da mesma forma, embora um estudo adequadamente dimensionado (com poder = 1 – β) minimize o risco de um erro tipo II (não detectar um efeito verdadeiro), nem os valores de p nem as considerações de poder garantem sozinhos resultados significativos na presença de vieses. De fato, erros do tipo I (α) e tipo II (β) são amplificados em contextos com múltiplos vieses ou quando vários estudos abordam a mesma questão, aumentando a probabilidade de resultados significativos falsos. A analogia com testes diagnósticos é esclarecedora; assim como sensibilidade e especificidade dependem da probabilidade pré-teste e da precisão do teste, os valores de p e o poder estatístico dependem do delineamento do estudo, do rigor metodológico e da ausência de vieses.^3^Essa compreensão está alinhada com a percepção de Ioannidis,^43^ que, por meio de modelagem matemática, concluiu que “a maioria dos achados de pesquisa publicados são falsos”. Seu artigo seminal enfatiza que metodologias falhas, baixas probabilidades pré-estudo das hipóteses testadas e vieses de publicação coletivamente corroem a confiabilidade de muitos achados relatados. Consequentemente, nenhuma única métrica – sejam valores de p, intervalos de confiança ou mesmo probabilidades posteriores Bayesianas – pode capturar completamente o verdadeiro valor de um resultado se os dados subjacentes ou as suposições não forem confiáveis.

Além das considerações metodológicas discutidas, é importante reconhecer que pressões externas – desde influências sociológicas e interesses econômicos a imperativos de *marketing* – também moldam a interpretação dos dados de ensaios clínicos. Esses fatores podem promover vieses sutis que levam a relatos seletivos e ao enquadramento dos resultados de maneira que favoreça certos desfechos, muitas vezes obscurecendo o verdadeiro valor clínico de uma intervenção. Embora uma exploração detalhada dessas influências esteja além do escopo da nossa revisão, reconhecer seu papel reforça a necessidade de uma avaliação crítica independente.

### Implicações para a prática clínica

A avaliação rigorosa de evidências clínicas não é meramente um exercício acadêmico – é fundamental para aprimorar a tomada de decisões e a educação clínica cotidiana. Em nossa prática, devemos aplicar consistentemente técnicas de avaliação crítica para dissecar dados de estudos, especialmente quando confrontados com reversões médicas, *spin* ou resultados divergentes.

Além disso, devemos sempre valorizar a promoção de uma cultura de debate e aprendizado contínuo sobre os fundamentos da Medicina Baseada em Evidências. Para traduzir essas percepções na prática clínica, o uso rotineiro de ferramentas padronizadas de avaliação de vieses, como a estrutura GRADE e a ferramenta de risco de viés Cochrane, pode ser muito útil.^44^

## Conclusão

A cardiologia contemporânea prospera em relação à avaliação rigorosa de evidências clínicas, mas permanece vulnerável às armadilhas do raciocínio mecanicista, *spin* e outras distorções da validade científica. Os exemplos discutidos – desde as reversões inesperadas vistas em estudos como o CAST até as manipulações sutis de desfechos e interpretações – enfatizam que mesmo pesquisas robustas podem ser suscetíveis a vieses e deficiências metodológicas. Resultados conflitantes, como os observados com a terapia MitraClip para regurgitação mitral funcional ou estratégias de anticoagulação para FA subclínica, acentuam a necessidade de superar o paradigma binário de “positivo vs. negativo” e adotar uma perspectiva mais contextualizada e sensível ao interpretar os dados. Os cardiologistas devem cultivar uma mentalidade vigilante, independente e crítica ao avaliar ensaios clínicos e diretrizes. Devemos também ter em mente que o método científico é um processo iterativo destinado a alcançar respostas válidas, em vez de uma verdade absoluta imutável; assim, nossas interpretações são provisórias e podem evoluir à medida que novas evidências surgem.
