# Supplementary material for: Medical Reversals, Spins e Resultados Divergentes em Ensaios na Cardiologia
Source: Arq Bras Cardiol. 2025 Jul 29;122(7):e20240884. [Article in Portuguese] doi: 10.36660/abc.20240884 (PMC12695093; doi:10.36660/abc.20240884)
Supplement: Translation

# Translation: Medical Reversals, Spins and Divergent Results in Cardiology Trials

**DOI:** 10.36660/abc.20240884i

**Authors:** José Nunes de Alencar, Bruno Robalinho Cavalcanti, Guilherme Augusto Teodoro Athayde

**Affiliations:** 1 Brazil Instituto Dante Pazzanese de Cardiologia, São Paulo, SP – Brazil; 2 Brazil Universidade Federal de Campina Grande, Campina Grande, PB – Brazil; 3 Brazil Dom José Maria Pires Metropolitan Hospital, João Pessoa, PB – Brazil

**Keywords:** Evidence-based Cardiology, Medical Reversals, Spin Bias

## Abstract

Cardiovascular medicine has witnessed remarkable breakthroughs, yet even highly regarded interventions can be undermined by flawed reasoning, excessive mechanistic assumptions, and the selective reporting of data. This article examines crucial pitfalls in contemporary cardiology, such as medical reversals, the impact of spin, and how bayesian methods can offer greater clarity in evaluating evidence, as they integrate prior knowledge with new data to generate more probabilistic, context-driven conclusions. This review advocates for a measured, critical approach to research appraisal, cautioning cardiologists against uncritically accepting trial conclusions at face value. Adopting this vigilant stance will help ensure that emerging therapies and interventions genuinely advance patient outcomes, guiding physicians toward more credible, transparent, and beneficial strategies in the ever-evolving field of cardiovascular medicine. Such vigilance is important to preserve the integrity of scientific inquiry and meaningful progress in patient care. This approach promotes reliability of published data.

## Introduction

Over the past decades, cardiovascular medicine has witnessed remarkable progress supported by rigorous clinical trials and comprehensive guidelines. Such advancements have unequivocally improved patient outcomes, reducing mortality and enhancing quality of life. Yet, this scientific evolution has not been free of missteps, controversy, and surprising turnarounds that challenge our preconceived notions of pathophysiology and the efficacy of certain interventions. Mechanical assumptions that once seemed self-evident have given way under the scrutiny of robust evidence, illustrating the hazards of relying on logic alone without testing hypotheses through rigorous study designs. Early interventions that promised improved survival based solely on mechanistic plausibility have at times led to unexpected clinical reversals once subjected to stringent investigation.^1,2^

The importance of critical appraisal and interpretation of clinical data in cardiology cannot be overstated. Clinicians must navigate an increasingly complex landscape of medical evidence, encompassing studies with conflicting findings, potential spin in reported outcomes, and evolving standards of methodological rigor. Assessing the validity, applicability, and clinical significance of new data has become an essential skill for the modern cardiologist.^3^ Such critical evaluation promotes tailored, individualized decision-making, whether considering the risks and benefits of coronary interventions, selecting candidates for catheter ablation of atrial fibrillation (AF), or determining when to initiate advanced heart failure (HF) therapies.^4^

This manuscript examines how reliance on untested mechanistic assumptions, susceptibility to spin, and challenges in interpreting complex or conflicting findings can compromise the integrity of evidence-based cardiology. Drawing upon emblematic cases, it highlights how medical reversals, endpoint manipulation, and the selective presentation of results may skew clinical decision-making (Central Illustration and Table 1).

**Table 1:** – Key Definitions: Medical Reversals, Spin, and Divergent Results

Term	Definition	Key Considerations/Examples
Medical Reversals	Occur when a widely adopted clinical intervention—often initially supported by observational studies or mechanistic rationale—is later refuted by robust randomized controlled trials.	For instance, the initial use of class IC antiarrhythmics to suppress ventricular arrhythmias was overturned by the CAST trial, demonstrating increased mortality.
Spin	Refers to the selective presentation or framing of study results to accentuate favorable outcomes while downplaying or obscuring adverse or neutral findings.	An example is the RITA-2 trial, where early symptomatic improvement was highlighted for percutaneous transluminal coronary angioplasty, despite the overall composite outcome favoring optimized medical therapy.
Divergent Results	Describe situations in which high-quality studies investigating the same clinical question yield conflicting outcomes, often due to differences in study design, populations, or endpoint definitions.	A notable example includes the contrasting results of the COAPT and MITRA-FR trials evaluating MitraClip therapy for functional mitral regurgitation.

### Not everything Is as it seems: medical reversals in cardiology

Cardiology is replete with studies that defy logical expectations and highlight how evidence-based medicine can overturn what once appeared to be indisputable truths.^5,6^ A striking example lies in the treatment of ventricular arrhythmias following acute myocardial infarction (MI). In the 1970s and 1980s, the advent of coronary care units dramatically reduced in-hospital mortality for MI patients.^7^ It then became apparent that many survivors later experienced ventricular arrhythmias, which were identified in several investigations as predictors of mortality.^8^ Drawing on this theoretical understanding, a team of researchers in the United States proposed that survival rates could be improved for patients with ischemic cardiomyopathy who were at elevated risk of death from ventricular arrhythmias if these irregular heartbeats were suppressed. The initial step in evaluating this hypothesis was the Cardiac Arrhythmia Pilot Study (CAPS), conducted in 1986. This study demonstrated that class IC antiarrhythmic medications, which function as sodium channel blockers, were capable of nearly eliminating ventricular arrhythmias in these individuals.^9^ With that, it seemed logical to add these agents to the therapeutic arsenal; if arrhythmias could be suppressed, mortality should be reduced.

However, the Cardiac Arrhythmia Suppression Trial (CAST) disrupted these assumptions. The study included 1,498 patients randomized to receive flecainide/encainide or placebo. After 10 months, the trial was halted due to excess deaths—especially arrhythmic deaths—in the group receiving the antiarrhythmics. This reversal became one of the most striking scientific about-faces of the era and sharply limited the use of class IC antiarrhythmics in patients with structural heart disease due to their proarrhythmic potential.^10^

Another unexpected finding was observed in the context of AF screening and treatment. AF is the most common sustained arrhythmia in clinical practice and is associated with mortality and thromboembolic events (TE), particularly stroke and HF. Furthermore, anticoagulation can significantly and safely reduce TE risk.^11^ It seemed self-evident that early identification and anticoagulation in AF patients would yield unequivocal benefits. Yet, the LOOP Study, published in 2021, challenged this notion.^12^ In this trial, 6,205 patients aged 70–90 years with at least one additional stroke risk factor were randomized to conventional management or continuous long-term subcutaneous electrocardiographic monitoring for AF detection. Patients with AF episodes ≥ six minutes were started on anticoagulation. After more than five years, AF was diagnosed three times more often in the monitored group, and anticoagulation was initiated more frequently. However, there were no differences in stroke or bleeding rates between the groups. The study’s results align with other trials, such as GUARD-AF, which also found no significant reduction in stroke rates with AF screening, suggesting that not all detected AF may be clinically significant enough to justify anticoagulation.^13^

One critique is that the LOOP Study population might not have been optimally selected to benefit from AF screening. The subgroup analyses suggested potential benefits in specific populations, such as those without prior cardiovascular disease, but these findings were not definitive and require further investigation.^14^ Moreover, concerns have been raised about the study’s methodology and the decision to start anticoagulation for AF episodes lasting only six minutes or more, as it remains uncertain whether such short-lived episodes require treatment.

Regarding another notable medical reversal, initial observational studies suggested that hormone replacement therapy (HRT) for women after menopause could lower cardiovascular risk. These studies indicated potential advantages, including decreased coronary heart disease (CHD) and reduced mortality rates.^15^ Randomized controlled trials, notably the Women’s Health Initiative (WHI),^16^ contradicted these findings by showing an increased risk of cardiovascular events, including CHD and stroke, in women receiving HRT.^16^ The WHI trials were stopped early due to these increased risks, leading to a significant shift in clinical practice away from using HRT for cardiovascular protection.^17^ More recent investigations have revealed that for women who entered menopause within the last 10 years, HRT offers therapeutic advantages without increasing cardiovascular dangers. Clinical trials^18^ and meta-analyses^19^ have provided evidence supporting these observations. This group falls within the “window of opportunity” for HRT prescription, which has been recommended by both international and national guidelines, particularly for women without a high risk of or previous cardiovascular events. These guidelines recommend that HRT be initiated within 10 years of menopause and/or before the age of 60 years; starting therapy after age 60 or more than 10 years after menopause may elevate the absolute risk of cardiovascular adverse events.^20,21^

### Distorted interpretations: spin in cardiovascular research

Some studies have seen their conclusions subtly distorted by the authors themselves, casting a more favorable light on a particular treatment than the data warrant. This practice, known as spin, can mislead readers and clinicians, potentially influencing therapeutic decisions.^22^

A notable example of spin can be found in the context of CAD. The RITA-2 trial, published in 1997, randomized 1,018 patients with stable angina to either balloon angioplasty (percutaneous transluminal coronary angioplasty, PTCA) or optimized medical therapy (OMT).^23^ The primary composite endpoint—death from any cause and nonfatal myocardial infarction (MI)—was assessed over five years (mean follow-up of 2.7 years). The results showed that PTCA increased the risk of the primary composite outcome compared with OMT, driven primarily by nonfatal MI. Although both groups initially experienced symptom improvement, statistical significance in favor of PTCA over OMT diminished after three years. The authors, however, emphasized the early symptomatic improvement in patients undergoing PTCA and suggested that clinicians weigh this benefit against a “small” excess procedural risk. Such framing did not accurately reflect the primary endpoint, where PTCA worsened outcomes. With a number needed to harm (NNH) of 33 over 2.7 years, the study’s “spin” diverted attention from the primary endpoint’s negative result.

A similar example emerged with the EXCEL trial, published in 2016, which evaluated percutaneous coronary intervention (PCI) with second-generation everolimus-eluting stents versus coronary artery bypass grafting (CABG) for left main coronary artery disease of low-to-intermediate anatomical complexity.^24^ The primary composite endpoint—death, MI, and stroke—was methodologically questionable. CABG is known to increase stroke risk compared to PCI, while PCI may be associated with higher rates of spontaneous MI. Combining these events into a single composite endpoint meant that the two interventions pulled the outcomes in opposite directions. Concerns also arose about the use of intention-to-treat analysis instead of per-protocol, given substantial crossover, and the evolving definitions of MI during the trial. Such changes may have favored PCI. As a result, a more transparent approach would separate out procedural MI and stroke as safety endpoints and focus solely on spontaneous MI as a primary outcome, as the NOBLE trial eventually did.^25^

Subgroup analyses represent another common avenue for spin. The ISIS-2 trial, a 2x2 factorial, randomized, placebo-controlled trial with over 17,000 patients, tested streptokinase and one month of aspirin at 162.5 mg/day in the setting of acute MI.^26^ It demonstrated a 20% reduction in vascular mortality with aspirin, 23% with streptokinase, and a 40% reduction with their combination over five weeks. The absolute risk reduction for aspirin alone was 2.4 deaths per 100 treated patients, corresponding to a number needed to treat (NNT) of 42. Despite these impactful results, journal editors at the time required subgroup analyses. In a deliberately ironic response, the authors reported outcomes by astrological signs, finding, for example, that patients born under Gemini and Libra did not seem to benefit from aspirin. This tongue-in-cheek demonstration underscored the folly of overreliance on arbitrary subgroup findings and emphasized the need to interpret these analyses with skepticism.^27^

Another subtle yet impactful means of shaping a trial’s narrative lies in changing the predefined endpoints or their definitions during the investigation. The ISCHEMIA trial is a prime example. This landmark study, costing approximately $100 million, was published in 2020 and enrolled 5,179 patients with moderate-to-severe ischemia, randomizing them to either OMT alone or OMT plus cardiac catheterization followed by PCI if indicated.^28^ Initially, the primary composite endpoint was cardiovascular death and MI. However, due to the lower-than-expected incidence of these events, the investigators added hospitalization and HF to the primary composite outcome.

While the investigators defined this modification *a priori*, the decision ultimately weakened the study’s interpretative clarity. Incorporating subjective endpoints such as unstable angina or HF hospitalizations along with objective outcomes such as death and MI diluted the clarity and reliance of the conclusions. The ISCHEMIA trial’s results demonstrated no significant difference between the invasive and conservative strategies, reinforcing the understanding that even in patients with moderate or severe ischemia, stable coronary artery disease often carries a favorable long-term prognosis.^29^

The selection of endpoints — and their underlying assumptions — can critically shape the outcomes of a trial. Consider the recently introduced OPTION trial, sponsored by the manufacturer of a device used for left atrial appendage closure (LAAC) alongside AF.^30^ OPTION compares AF ablation plus concomitant LAAC against AF ablation plus continued direct oral anticoagulants. At first glance, the trial’s aim—potentially reducing or eliminating the need for long-term anticoagulation—may appeal to patients and clinicians. However, a closer look at OPTION’s design raises multiple red flags. The trial employs a noninferiority framework and includes all-cause mortality, an endpoint known to remain unaffected by both ablation and LAAC, as part of a composite efficacy measure. This approach, coupled with a relatively small sample size and a noninferiority margin chosen under optimistic event-rate assumptions, makes it easier for LAAC to achieve noninferiority without demonstrating any meaningful advantages in preventing stroke or systemic embolism. Further concerns arise from the choice of conducting the primary analysis in the intention-to-treat population—less appropriate for noninferiority studies — and excluding procedural bleeding from the primary safety endpoint, thus underestimating the true procedural risks.^31^

## Reconciling divergent findings in clinical trials

Sometimes, high-quality clinical trials yield conflicting outcomes on the same medical issue, making it difficult for healthcare professionals to determine the best way to apply these findings in practice.

A representative case involves the percutaneous repair of secondary mitral regurgitation using the MitraClip device. Two landmark trials, COAPT^15^ and MITRA-FR,^16^ both published in 2018, examined the clinical impact of adding MitraClip to guideline-directed medical therapy in patients with functional mitral regurgitation. While COAPT focused on HF hospitalization at one year, MITRA-FR assessed the composite of death and HF hospitalization over a similar time frame. Despite similar methodologies, the outcomes diverged markedly. In COAPT, MitraClip significantly reduced hospitalizations and overall mortality, whereas MITRA-FR showed no improvement. When clinical trials yield divergent results, it is essential to conduct a thorough analysis to understand the reasons underlying these discrepancies. Several key factors should be examined:

1. **Study design and population**: Differences in study design, such as inclusion and exclusion criteria, can lead to variations in patient populations. For instance, COAPT and MITRA-FR had different criteria for the severity of mitral regurgitation and left ventricular function, which significantly influenced their outcomes.^32^
2. **Outcome definitions**: Variations in how primary and secondary outcomes are defined and measured can lead to different interpretations of efficacy. The COAPT trial had more stringent criteria for procedural success and durability of mitral regurgitation reduction compared to MITRA-FR.^32^
3. **Treatment protocols**: Differences in the implementation of treatment protocols, including the use of guideline-directed medical therapy, can affect trial outcomes. COAPT ensured patients were on maximally tolerated medical therapy before enrollment, which was not as rigorously enforced in MITRA-FR.^32^
4. **Center and Operator Experience**: The experience of the centers and operators performing the interventions can impact procedural success rates and outcomes. COAPT was conducted in centers with significant experience in transcatheter mitral valve repair, which may have contributed to its positive results.^32^
5. **Statistical Analysis and Interpretation**: The statistical methods used to analyze data, including handling of missing data and adjustments for multiple comparisons, can influence the results.^33^

A similar challenge emerged in 2023 with two trials evaluating anticoagulation for subclinical AF detected by implantable cardiac devices. NOAH-AFNET 6 included 2,356 patients aged ≥65 years with one or more episodes of ≥6 minutes of subclinical AF plus an additional thromboembolic risk factor, randomizing them to placebo or edoxaban.^34^ The primary endpoint was a composite of cardiovascular death, stroke, or systemic embolism. ARTESIA, enrolling 4,012 patients with at least one ≥6-minute (but ≤24-hour) subclinical AF episode, randomized them to apixaban or aspirin (81 mg) and assessed the incidence of stroke or systemic embolism.^35^ NOAH-AFNET 6 stopped early for futility, with no difference in the primary endpoint and increased bleeding in the edoxaban arm. In ARTESIA, apixaban reduced TE but at the cost of higher bleeding rates. Examining potential causes of this divergent result, we can conclude that:

1. **Study design and population**: NOAH-AFNET 6 focused on patients with atrial high-rate episodes detected by cardiac implantable electronic devices, using edoxaban as the anticoagulant. ARTESIA evaluated apixaban in a similar population but included patients with additional risk factors for stroke, such as a higher prevalence of vascular disease. The ARTESIA trial also had a larger sample size, enrolling 4012 patients compared to 2534 in NOAH-AFNET 6.^36^
2. **Outcome definitions**: Both trials defined their primary efficacy outcome as a composite of stroke, systemic embolism, MI, pulmonary embolism, or cardiovascular death. However, ARTESIA included transient ischemic attack with diffusion-weighted MRI evidence of cerebral infarction as part of its primary outcome, which was not explicitly mentioned in NOAH-AFNET 6.^37^
3. **Treatment protocols**: The treatment protocols differed in the choice of anticoagulant—edoxaban in NOAH-AFNET 6 and apixaban in ARTESIA. NOAH compared edoxaban to placebo, but ARTESIA specifically used aspirin as the comparator.

When data are divergent, a meta-analysis might be helpful. A meta-analysis of the two trials demonstrated that oral anticoagulation reduced ischemic stroke risk (RR 0.68, 95% CI 0.50-0.92) and increased major bleeding risk (RR 1.62, 95% CI 1.05-2.50). The meta-analysis also highlighted a low heterogeneity (I^2^ = 0%), indicating consistency in the findings across the trials.^38^

Another approach to reconcile divergent findings and enhance the interpretation of clinical trials lies in Bayesian reasoning. Bayesian methods integrate prior beliefs with newly acquired evidence to generate posterior probabilities, providing a more intuitive and clinically relevant interpretation than traditional frequentist statistics. By focusing on the probability that a given intervention is truly beneficial, rather than relying solely on p-values or binary significance thresholds, Bayesian thinking permits a more flexible and context-sensitive assessment of evidence.^3^

Bayesian statistics begin with a prior probability that a given treatment is effective.^39^ In the setting of a randomized clinical trial comparing a new therapy (T) to a control (C), the initial assumption might be one of equipoise: P(T > C) = 0.5. After collecting and analyzing the trial’s data, the observed results are combined with this prior to yield a posterior probability. If the posterior probability that T is superior to C surpasses a certain threshold (e.g., 0.975), one can be more confident that the intervention is genuinely effective.^40^ Alternatively, if the data suggest no meaningful advantage, the Bayesian framework readily expresses how these results modify one’s belief in the treatment’s efficacy. Unlike frequentist methods, where a 95% confidence interval does not assign a probability to the parameter of interest, the Bayesian 95% credible interval provides a direct probability statement about the parameter.^41^ Instead of stating that an outcome is statistically significant, Bayesian methods allow clinicians to discuss the probability that a given difference in outcomes (e.g., a reduction in MI or stroke) is real and clinically relevant. In this way, Bayesian reasoning moves beyond p-values toward more actionable interpretations that can guide patient-centered decision-making.

A good example of how Bayesian analysis can offer a distinct interpretation of results is its application in the MINT trial. This study included 3,504 patients with MI and anemia (hemoglobin <10 mg/dL), who were randomized between restrictive transfusion strategies—transfusing only when hemoglobin levels were below 7-8 mg/dL—and liberal strategies (transfusing when hemoglobin <10 mg/dL). The primary outcome, a composite of MI and death within 30 days, was similar between groups, with a confidence interval of 0.99 to 1.34; p=0.07. Despite this, there was a strong trend toward a higher number of events in the restrictive group (which might have been confirmed with a larger sample size), including both death and MI, coupled with a very low rate of adverse events associated with transfusion. In light of these findings, analyzing the results from the perspective of the benefit-risk balance of the strategy in this population—based on existing knowledge—is crucial to identifying subgroups of patients who might benefit from transfusion, even in the context of a “negative” study.^42^

In traditional frequentist analyses, statistical significance is often determined by whether a p-value is less than 0.05. Such an approach can lead to binary, “positive vs. negative” interpretations, neglecting the entire probability distribution of the true effect. Similarly, while an adequately powered study (with power = 1 – β) minimizes the risk of a Type II error (failing to detect a true effect), neither p-values nor power considerations alone guarantee meaningful results in the presence of bias. In fact, type I (α) and type II (β) errors are amplified in settings with multiple biases or when numerous studies address the same question, increasing the likelihood of spurious significant findings. The analogy with diagnostic tests is illuminating just as sensitivity and specificity depend on the pre-test probability and test accuracy, so do p-values and power depend on study design, methodological rigor, and absence of bias.^3^ This recognition aligns with the insight provided by Ioannidis,^43^ who, through mathematical modeling, concluded that “most published research findings are false”. His seminal article emphasizes that flawed methodologies, low pre-study probabilities of tested hypotheses, and publication biases collectively erode the trustworthiness of many reported findings. Consequently, no single metric – be it p-values, confidence intervals, or even Bayesian posterior probabilities – can fully capture the true value of a result if the underlying data or assumptions are unsound.

In addition to the methodological considerations discussed, it is important to recognize that external pressures – ranging from sociological influences and economic interests to marketing imperatives – also shape the interpretation of clinical trial data. These factors can foster subtle biases that lead to selective reporting and the framing of results in a way that favors certain outcomes, often obscuring the true clinical value of an intervention. While a detailed exploration of these influences is beyond the scope of our review, acknowledging their role reinforces the necessity for an independent, critical appraisal.

### Implications for clinical practice

The rigorous evaluation of clinical evidence is not merely an academic exercise – it is central to enhancing everyday clinical decision-making and education. In our practice, we must consistently apply critical appraisal techniques to dissect trial data, particularly when confronted with medical reversals, spin, or divergent outcomes.

Moreover, we must always place a high value on promoting a culture of debate and continuous learning regarding the fundamentals of Evidence Based Medicine. Additionally, to further translate these insights into clinical practice, the routine use of standardized bias assessment tools, such as the GRADE framework and the Cochrane Risk of Bias Tool could be useful.^44^

## Conclusion

Contemporary cardiology thrives on the rigorous evaluation of clinical evidence, yet it remains vulnerable to the pitfalls of mechanistic reasoning, spin, and other misrepresentations of scientific validity. The examples discussed – from the unexpected reversals seen in trials like CAST to the subtle manipulations of endpoints and interpretations – emphasize that even robust research can be susceptible to biases and methodological shortcomings. Conflicting results, such as those observed with MitraClip therapy for functional mitral regurgitation or anticoagulation strategies for subclinical AF, accentuate the need to move beyond a binary “positive vs. negative” paradigm and instead adopt a nuanced, context-sensitive perspective when interpreting data. Cardiologists must cultivate a vigilant, independent, and critical mindset when assessing clinical trials and guidelines. We must also have in mind that scientific method is an iterative process aimed at achieving valid answers rather than an immutable absolute truth; thus, our interpretations are provisional and may evolve as further evidence emerges.
